# *SIRT1* (rs3740051) role in pituitary adenoma development

**DOI:** 10.1186/s12881-019-0892-x

**Published:** 2019-11-20

**Authors:** Rasa Liutkeviciene, Alvita Vilkeviciute, Greta Morkunaite, Brigita Glebauskiene, Loresa Kriauciuniene

**Affiliations:** 1 Neuroscience Institute, Lithuanian University of Health Sciences, Medical Academy, Eivenių 2, LT-50161 Kaunas, Lithuania; 2 Department of Ophthalmology, Lithuanian University of Health Sciences, Medical Academy, LT-50161 Kaunas, Lithuania; 3 Lithuanian University of Health Sciences, Medical Academy, LT-50161 Kaunas, Lithuania

**Keywords:** Pituitary adenoma, *SIRT1* (rs4746720, rs3740051) gene polymorphisms, Invasiveness

## Abstract

**Background:**

Our purpose was to determine if *SIRT1* (rs4746720, rs3740051) genotypes have an influence on the development of pituitary adenoma (PA).

**Methods:**

The study group included 142 patients with pituitary adenoma (PA) and the control group consisted of 826 healthy people. The genotyping of *SIRT1* (rs4746720, rs3740051) was carried out using the real-time polymerase chain reaction method.

**Results:**

Statistically significant results were obtained in the analysis of *SIRT1* rs3740051. Significant differences in genotype (G/G, G/A, A/A) distribution were obtained comparing patients with PA without recurrence and PA with recurrence (0, 17.9, 82.1% vs. 6.7, 6.7, 86.7%, respectively, *p* = 0.022). Also, statistically significant differences were observed when comparing the genotype (G/G, G/A, A/A) distribution in the non-invasive PA group and the invasive PA group (3.4, 25.9, 70.7% vs. 0, 8.3, 91.7%, respectively, *p* = 0.003), and allele G was less frequently observed in invasive PA, than in non-invasive PA (4.2% vs. 16.4%, *p* < 0,001). Further analysis revealed that G/A (OR = 0.261; 95% CI:0.099–0.689; *p* = 0.007) and each allele A (OR = 0.229; 95% CI:0.091–0.575; *p* = 0.002) were associated with lower odds of occurring an invasive PA.

**Conclusions:**

Our study revealed that *SIRT1* rs3740051 is associated with PA recurrence and invasiveness. The haplotype containing alleles C-A in rs12778366-rs3740051 was found to be associated with increased odds of PA development as well.

## Background

Pituitary adenoma (PA), a benign pituitary tumour, originates in the adenohypophyseal cells of the anterior lobe of the pituitary gland [1]. Usually, there is no capsule to isolate this soft tumour from microglia and the surrounding structures can be infiltrated by the growing tumour [2]. According to their size PAs can be classified as microadenomas and macroadenomas [2]. Women are at a twice higher risk of developing PA in comparison with men [1]. Meta-analysis showed that pituitary tumours are common and occur in almost 20% of the population [3]; they amount to about 10 to 15% of surgically removed intracranial tumours [4–6]. A study by Daly et al. revealed that the prevalence of PAs is one case in 1064 individuals [7]. Few studies have found age-adjusted incidence rate of PA is 2.7–2.87 cases per 100,000 [8, 9]. Hemminki and colleagues have analyzed familial risks for PAs and revealed that the risk of PA was significantly increased in individuals whose siblings were diagnosed with colorectal cancer [10].

However, the mechanisms of PA development are still not clear, as sporadic pituitary tumours rarely involve mutations of classical oncogenes or tumour suppressor genes [4, 5]. Despite scarcely being malignant, pituitary adenomas may display various invasive behaviors that have been related to the particular pathological subtype [11] and to different degrees of morbidity [3]. The tumour invasiveness affects the management and prognosis of PA and needs to be investigated [12]. The present study aimed to identify a molecular marker possibly involved in PA tumorigenesis, which could be used as a potential diagnostic and prognostic tool. In our study, two SIRT1 gene polymorphisms were selected for further investigation.

Sirtuins (SIRT) are a highly conserved family of NAD-dependent class III deacetylases that help to regulate the longevity of various organisms [13]. In mammals, seven human Sir2 homologues (sirtuins) designated as SIRT1 to SIRT7 have been identified to date. These are associated with calorie restriction, aging, metabolism, stress response, inflammation, cancer, transcriptional silencing, chromosomal stability, cell differentiation, apoptosis, and DNA repair. Generally, sirtuins are reported to have key roles in cellular senescence, cell differentiation, and inflammation [14–17]. It has been determined that levels of *SIRT1* increased significantly in hepatocellular carcinoma [18], breast cancer [19], glioblastoma [20], lymphoma [21], and other types of cancer development and invasion [22–25]. The *SIRT1* rs12778366 polymorphism was found to have a relation with breast cancer [26]. In our previous study, analyzing the *SIRT1* rs12778366 polymorphism in the overall group, we revealed different genotype distributions in the PA and control groups [27]. That is why we aimed to investigate whether there was an association between pituitary adenoma and two other polymorphisms (rs4746720, rs3740051) of the *SIRT1* gene.

## Methods

### Patients and selection

The study was carried out in the Department of Ophthalmology, Department of Pathology and in the Department of Neurosurgery, Lithuanian University of Health Sciences (LUHS).

Our study consisted of 142 patients with pituitary adenoma (PA) group and controls who were involved by the inclusion and exclusion criteria [28].

### Activeness and recurrence evaluation

The analysis of all pituitary adenomas was based on histopathological findings of PA and hormone levels in the blood serum before surgery. All 142 subjects were categorized into two groups – active and inactive PA. The active PA group was not divided into smaller subgroups by increase of specific hormone because dominant tumours were prolactinomas, while others would not make sufficient numbers for the study. Since some of the 142 subjects had already had surgery in recent years, we categorized them by recurrence of pituitary adenoma into two groups – PA with and without recurrence.

Patients before medical or surgical treatment who were newly diagnosed with PA or came for follow up with PAs diagnosis to the Department of Neurosurgery were included in the study which lasted for 5 years and was financed by the Research Council of Lithuania (grant no. MIP 008/2014). Pituitary adenoma recurrence was diagnosed when enlargement of a residual tumor or a new growth was documented on follow-up magnetic resonance imaging (MRI) after surgical resection during the period of this study. The residual tumor was considered stable if there no signs of tumor progression on follow-up MRI. Most of prolactinomas were surgically treated because of remaining pressure effects of surrounding structures or because of ineffective medical treatment.

### Invasiveness evaluation, Ki-67 labelling index, DNA extraction and genotyping

The analysis of pituitary adenoma invasiveness and method for Ki-67 labelling index has been widely described in our previous article [28], as well as DNA extraction and genotyping based on real-time polymerase chain reaction (RT-PCR) methods [29].

### Statistical analysis

Deviation from Hardy-Weinberg equilibrium (HWE) of *SIRT1* (rs3740051) alleles was calculated with the Pearson’s χ2 statistical test in both the case and the control subjects.

Statistical analysis was performed using SPSS 20.0 software (IBM SPSS, Armonk, NY, USA).

Frequencies of *SIRT1* (rs3740051) genotypes and alleles, and other categorical variables were expressed as absolute numbers with percentages in brackets compared using the Pearson’s χ2 and Fisher’s exact test (when N is less than 50) in study groups. The computational of statistical power was performed using Bioinformatics Institute’s Online Sample Size Estimator (OSSE) (http://osse.bii.a-star.edu.sg/calculation2.php). The age was presented as median and interquartile range (IQR) and compared between PA and control groups using nonparametric Mann-Whitney U test. Binomial logistic regression analysis was performed to estimate the impact of genotypes on PA development and expressed as genetic models (codominant: heterozygotes versus wild type homozygotes and minor allele homozygotes versus wild type homozygotes; dominant: minor allele homozygotes and heterozygotes versus wild type homozygotes; recessive: minor allele homozygotes versus wild type homozygotes and heterozygotes; overdominant: heterozygotes versus wild type homozygotes and minor allele homozygotes; additive model was used to evaluate the impact of each minor allele on PA development). Odds ratios (ORs) with 95% confidence intervals (CIs) are presented. Akaike information criterion (AIC) with the lowest AIC values showed the best genetic models. Haplotypes were constructed of two single nucleotide polymorphisms (SNPs) located on the same chromosome (rs12778366-rs3740051). Estimation of haplotype association tests was carried out using PLINK software version 1.07 [30] as reported previously [29]. Statistical significance was indicated when *p* < 0.05.

## Results

The study group involved 142 patients with pituitary adenoma (PA): 55 males and 87 females. The median age of the groups was 53.5 years. The control group consisted of 826 subjects: 320 males and 506 females; the median age was 53 years as well and did not differ statistically significantly from PA group (Table 1). The distributions of PA recurrence, invasiveness, hormonal activity, and Ki-67 LI are presented in Table 1.

**Table 1:** Demographic characteristics

Characteristic	Group		*p* value
	PA, N (%) (*n* = 142)	Control, N (%) (*n* = 826)	
Gender			
Females	87 (61.3)	506 (61.3)	0.998^*^
Males	55 (38.7)	320 (38.7)	
Age, median (IQR)	53.5 (22)	53 (11)	0.764^**^
Recurrence			
Yes	30 (21.1)	–	–
No	112 (78.9)		
Invasiveness			
Yes	84 (59.2)	–	–
No	58 (40.8)		
Hormonal activity			
Yes	80 (56.3)	–	–
No	62 (43.7)		
Ki-67 LI			
< 1%	41 (74.6)		
1%	8 (14.5)		
> 1%	6 (10.9)		

*IQR* interquartile range

^*^Pearson’s χ 2 test

^**^Mann-Whitney U test

### The frequency of genotypes and alleles of rs3740051 in patients with PA and control subjects

While the *SIRT1* rs4746720 polymorphism was not involved in statistical analysis because genotyping results showed only wild type genotype for all study subjects, the genotyping of *SIRT1* rs3740051 was performed successfully and the genotype distribution did not deviate from Hardy–Weinberg equilibrium (HWE) (*p* > 0.05).

Analysis of genotype (G/G, G/A and A/A) and allele (A and G) distributions in PA and control groups did not reveal any statistically significant differences between these groups (83.1, 15.5 and 1.4% vs. 86.6, 12.5 and 1%, *p* = 0.534; 90.8 and 9.2% vs. 92.8 and 7.2%, *p* = 0.248, respectively) (Table 2).

**Table 2:** The frequency of genotypes and alleles of rs3740051 in patients with PA and control subjects

Genotype/allele	Frequency (%)				
	Control group, N (%) (*n* = 826)	HWE *p* value	PA group, N (%) (*n* = 142)	HWE value	*p* value^*^
Genotype					
G/G	8 (1.0)	0.053	2 (1.4)	0.414	0.534
G/A	103 (12.5)		22 (15.5)		
A/A	715 (86.6)		118 (83.1)		
Allele					
G	119 (7.2)		26 (9.2)		0.248
A	1533 (92.8)		258 (90.8)		

*PA* pituitary adenoma, *p value* significance level, *HWE p value* Hardy-Weinberg significance level

^*^Pearson’s χ 2 test

Binary logistic regression was performed to assess the rs3740051 impact on PA development, but the results did not show any significance (Additional file 1).

### The frequency of genotypes and alleles of rs3740051 in patients with PA and control subjects by gender

Statistical analysis was performed in patients and control subjects by gender as well. Unfortunately, the results did not reveal any associations of the rs3740051 with PA neither in males nor in females (Additional files 2, 3).

Looking for the possible cause of PA development, we performed a detail statistical analysis of the frequency of genotypes and alleles of rs3740051 by PA recurrence, invasiveness, and hormonal activity.

### The frequency of genotypes and alleles of rs3740051 in patients with PA by recurrence

Statistical analysis revealed significant differences in genotype (G/G, G/A, A/A) distribution only between the PA without recurrence and PA with recurrence groups (0, 17.9, 82.1% vs. 6.7, 6.7, 86.7%, *p* = 0.022 with the lack of power (3.4%). Logistic regression analysis did not show any significant results (Table 3).

**Table 3:** The frequency of genotypes and alleles of rs3740051 in patients with PA without recurrence and PA with recurrence

Genotype/allele	Frequency (%)			OR^***^ (CI) *p* value
	PA without recurrence group, N (%) (*n* = 112)	PA with recurrence group, N (%) (*n* = 30)	*p* value^*^	
Genotype				
G/G	0 (0)	2 (6.7)		–
G/A	20 (17.9)	2 (6.7)	0.022^**^	0.354 (0.078–1.614) 0.35
A/A	92 (82.1)	26 (86.7)		Reference
Allele				
G	20 (8.9)	6 (9.7)	0.856^*^	1.124 (0.444–2.843) 0.805
A	204 (91.1)	56 (90.3)		–

*PA* pituitary adenoma, *p value* significance level, *OR* odds ratio, *CI* confidence interval.

^*^Pearson’s χ 2 test

^**^Fisher’s exact test

^***^Odds ratios were evaluated under the codominant and additive genetic models

Bold entries have significant values

### The frequency of genotypes and alleles of rs3740051 in patients with PA by PA invasiveness

The next part of our study was to evaluate the impact of rs3740051 on PA development by tumour invasiveness. Statistically significant differences were observed when comparing the genotype (G/G, G/A, A/A) distribution in the non-invasive PA group and the invasive PA group (3.4, 25.9, 70.7% vs. 0, 8.3, 91.7%, respectively, *p* = 0.003) (Table 4). Also, allele G was less frequently observed in invasive PA, than in non-invasive PA (4.2% vs. 16.4%, *p* < 0,001) with the statistical power of 65.6%. Logistic regression analysis showed that genotype G/A (OR = 0.261; 95% CI:0.099–0.689; *p* = 0.007) and each allele A (OR = 0.229; 95% CI:0.091–0.575; *p* = 0.002) were associated with lower odds of occurring an invasive PA (Table 4).

**Table 4:** The frequency of genotypes and alleles of rs3740051 in patients with non-invasive PA and invasive PA

Genotype/allele	Frequency (%)			OR^**^ (CI) *p* value
	Non-invasive PA group, N (%) (*n* = 58)	Invasive PA group, N (%) (*n* = 84)	*p* value^*^	
Genotype				
G/G	2 (3.4)	0 (0)		**–**
G/A	15 (25.9)	7 (8.3)	0.003^*^	0.248 (0.094–0.658) 0.005
A/A	41 (70.7)	77 (91.7)		Reference
Allele				
G	19 (16.4)	7 (4.2)	< 0.001^*^	0.229 (0.091–0.575) 0.002
A	97 (83.6)	161 (95.8)		

*PA* pituitary adenoma, *p value* significance level, *OR* odds ratio, *CI* confidence interval.

^*^Pearson’s χ 2 test

^**^Odds ratios were evaluated under the codominant and additive genetic models

### The frequency of genotypes and alleles of rs3740051 in patients with PA by the PA hormonal activity

The distribution of genotypes and alleles of rs3740051 was evaluated in patients with active PA and non-active PA. Unfortunately, we found no associations between the rs3740051 and active PA or non-active PA development (Additional file 4). Also, we compared the distribution of genotypes and alleles of rs3740051 between patients with prolactinomas as those were the dominant tumours and control group, but no statistically significant differences were found (Additional file 5). Logistic regression analysis did not show any significant results as well (Additional file 6).

### Ki-67 labeling index and *SIRT1* rs3740051 association in patients with PA

Ki-67 LI was evaluated in 55 patients with PA (Table 5). The analysis of *SIRT1* rs3740051 genotypes was performed in patients with PA by Ki-67 LI. According to our results, the rs3740051 G/G genotype was not observed in this group of patients. Also, we compared the distribution of A/A and G/A genotypes in all three Ki-67 LI groups, but results did not show statistically significant differences (Table 5).

**Table 5:** Association of Ki-67 LI and rs3740051 in patients with PA

Genotype of rs3740051	Ki-67 LI			p^*^ value
	< 1%	1%	> 1%	
G/G, n (%)	0 (0)	0 (0)	0 (0)	0.534
G/A, n (%)	5 (12.2)	0 (0)	1 (16.7)	
A/A, n (%)	36 (87.8)	8 (100)	5 (83.3)	

*p value* significance level

^*^Pearson’s χ2 test,

### Haplotype associations with PA development

In this section, we performed an association analysis between the risk of PA and the haplotype of rs12778366-rs3740051. This haplotype was constructed from one previously published SNP (rs12778366) [27] and one SNP analysed in our study (rs3740051). We evaluated the linkage disequilibrium between the two rs12778366-rs3740051 and obtained a D′ value of 0.196.

Only the haplotype containing alleles C-A in rs12778366-rs3740051 was significantly associated with the increased risk of PA development (17.56% in PAs vs. 11.22% in control subjects, χ2 = 8.984, *p* = 0.003).

## Discussion

SIRT1 is a conserved nicotinamide adenine dinucleotide-dependent protein deacetylase that acts as a longevity regulator [31]. However, SIRT1 is also associated with cancer cell growth, apoptosis, and tumorigenesis [32]. It has been hypothesized that SIRT1 inactivates the Akt pathway in a SIRT1 deacetylase-dependent manner; thus, SIRT1 is responsible for the deacetylation of the tumour suppressor PTEN [33], a known negative regulator for the phosphatidylinositol 3-kinase (PI3K)/Akt pathway, which is a key oncogenic pathway that promotes cell growth and survival. It has been suggested that modulation of mammalian sirtuins like SIRT1 may thus be a right approach in slowing the trajectory of aging-related degenerative changes in the central nervous system and neurologic disorders [34]. SIRT1 was first found as a nuclear protein [35] that may also shuttle to the cytoplasm during neuronal differentiation and neurite outgrowth [36–38], tumour progression [39, 40] and apoptosis [41]. Roth and Chen [42] suggested that SIRT1 may play a protective role in normal cell cycle by suppressing the tumorigenesis. While it was proposed that tumor cells act through SIRT1-regulated pathways by preventing aberrant life cycle, the main role of SIRT1 remains unclear.

Several studies have analysed SIRT1 gene polymorphisms and expression in association with various types of tumours. Rizk et al [26] have investigated *SIRT1* (SNPs) rs3758391, rs3740051, and rs12778366 gene polymorphisms in patients with breast cancer and proved that two of those (rs3758391 and rs12778366) were associated with breast cancer development in the Egyptian population. Also, their data implicate that *SIRT1* rs3740051 can be a possible contributor to breast tumorigenesis and suggest that the G allele may play a role in the increased expression of SIRT1 and higher susceptibility to breast cancer [26].

Immunohistochemical analysis has shown that SIRT1 is significantly elevated in human prostate cancer [19], acute myeloid leukemia [43], primary colon [44], and different types of skin cancer [45], but Wang et al*.* have found reduced SIRT1 expression in several types of cancer (glioblastoma, bladder carcinoma, prostate carcinoma, ovarian cancer, and hepatic carcinoma), compared to the corresponding normal tissues [46]. Chu-Xia Deng also concluded that the SIRT1 acts as a tumour suppressor rather than a promoter [47].

Our previous study analyzed other SIRT1 SNP and demonstrated that SIRT1 rs12778366 could be associated with PA development. We previously found that the carriers of the minor allele C at *SIRT1* rs12778366 had an increased risk of PA development [27]. Our current study revealed that the *SIRT1* rs3740051 A/A genotype decreases odds of recurrent PA development, and the G/A and G/G genotypes and each G allele increases the odds of non-invasive PA development. To our knowledge, it is the first study analysing the *SIRT1* rs3740051 gene polymorphism association with PA. We disagree with the Rizk et al [26] study, which states that the G allele is associated with the increased expression of SIRT1 and higher susceptibility to breast cancer [26], as we believe that the G allele increases the odds of non-invasive PA development. Also, we found that the haplotype containing alleles C-A in rs12778366-rs3740051 was significantly (*p* = 0.003) associated with increased risk of PA development in our study.

To our knowledge, the frequency of pituitary adenomas varies greatly according to age and sex. The various adenoma types have their peak occurrence in distinctly different age groups and differ greatly in their female-to-male ratios [48]. The biology and the clinical course of clinically non-functioning pituitary adenoma seem to differ in women and men [49]. Unfortunately, our study results did not reveal any associations of rs3740051 with PA neither in males nor in females. In an analysis of males and females separately, we expected to find differences of SNPs between patients with PA and controls, and a possible link to PA in females. In our previous study [27] significant differences comparing *SIRT1* rs12778366 genotype distribution in females with PA with healthy females were revealed. The T/C genotype was less frequently present in females with PA compared with the healthy control females (0 vs. 17.5%, respectively; *p* < 0.001) and C/C was more frequent in PA females compared with healthy females (19.3 vs. 2.7%, respectively; *p* < 0.001). When analyzing genotype distribution in males, T/C genotype was not observed in males with PA, only in healthy control males (0 vs. 17.4%, respectively; *p* < 0.001), while the C/C genotype was more frequent in males with PA compared with the controls (18.2 vs. 2.0%, respectively; *p* < 0.001). Our present study showed that *SIRT1* rs3740051 SNP decreases the odds of recurrence and increases the odds of non-invasive PA development as well.

## Conclusions

Our study revealed that *SIRT1* rs3740051 is associated with PA recurrence and invasiveness. The haplotype containing alleles C-A in rs12778366-rs3740051 was found to be associated with increased odds of PA development as well.

## Supplementary information

**Additional file 1.** The impact of rs3740051 on PA development. Logistic regression analysis was performed to evaluate the impact of rs3740051 on PA development under genetic models.

**Additional file 2.** The frequency of genotypes and alleles of rs3740051 in patients with PA and control subjects by gender. Frequency of genotypes and alleles of rs3740051 were estimated to compare differences between patients with PA and control subjects by gender.

**Additional file 3.** The impact of rs3740051 on PA development by gender. Logistic regression analysis was performed to evaluate the impact of rs3740051 on PA development under genetic models in female and male groups.

**Additional file 4.** The frequency of genotypes and alleles of rs3740051 in patients with active PA and inactive PA. Frequency of genotypes and alleles of rs3740051 were estimated to compare differences between patients with active PA and inactive PA.

**Additional file 5.** The frequency of genotypes and alleles of rs3740051 in patients with prolactinomas and control subjects. Frequency of genotypes and alleles of rs3740051 were estimated to compare differences between patients with prolactinomas and control subjects.

**Additional file 6.** The impact of rs3740051 on development of prolactinomas. Logistic regression analysis was performed to evaluate the impact of rs3740051 on prolactinomas development under genetic models.

## Data Availability

The datasets used and analysed during the current study are available from the corresponding author on reasonable request.

## Abbreviations

A: Adenine
AIC: Akaike information criterion
Akt: Serine/threonine-protein kinase
C: Cytosine
CIs: Confidence intervals
DNA: Deoxyribonucleic acid
G: Guanine
HWE: Hardy-Weinberg equilibrium
IQR: Interquartile range
Ki-67 LI: Ki-67 labelling index
LUHS: Lithuanian University of Health sciences
MRI: Magnetic resonance imaging
NAD: Nicotinamide adenine dinucleotide
OR: Odds ratios
OSSE: Online sample size estimator
*p* value: Significance level
PA: Pituitary adenoma
PI3K: Phosphatidylinositol 3-kinase
PTEN: Phosphatase and tensin homologue
RT-PCR: Real-time polymerase chain reaction
SIRT: Sirtuin
SNP: Single nucleotide polymorphism
T: Thymine
