# Supplementary material for: SIRT1 (rs3740051) role in pituitary adenoma development
Source: BMC Med Genet. 2019 Nov 20;20:185. doi: 10.1186/s12881-019-0892-x (PMC6868839; doi:10.1186/s12881-019-0892-x)
Supplement: Supplementary file 1 — Additional file 1. The impact of rs3740051 on PA development. Logistic regression analysis was performed to evaluate the impact of rs3740051 on PA development under genetic models. [file 12881_2019_892_MOESM1_ESM.docx]

***Additional file 1. The impact of rs3740051 on PA development***

| **Model** | **Genotype/allele** | **OR (95 % CI)** | **p value** | **AIC** |
| --- | --- | --- | --- | --- |
| Codominant | G/A vs. A/A  G/G vs. A/A | 1.307 (0.793;2.155)  1.347 (0.287;6.309) | 0.294  0.706 | 810.021 |
| Dominant | G/A+G/G vs. A/A | 1.310 (0.809;2.122) | 0.272 | 808.022 |
| Recessive | G/G vs. G/A+A/A | 1.297 (0.277;6.065) | 0.741 | 809.077 |
| Overdominant | G/A vs. G/G+A/A | 1.301 (0.790;2.145) | 0.301 | 808.155 |
| Additive | G | 1.261 (0.824;1.930) | 0.285 | 808.090 |

OR – odds ratio, CI – confidence interval, AIC-akaike information criteria, p-significance level.
