# Supplementary material for: SIRT1 (rs3740051) role in pituitary adenoma development
Source: BMC Med Genet. 2019 Nov 20;20:185. doi: 10.1186/s12881-019-0892-x (PMC6868839; doi:10.1186/s12881-019-0892-x)
Supplement: Supplementary file 2 — Additional file 2. The frequency of genotypes and alleles of rs3740051 in patients with PA and control subjects by gender. Frequency of genotypes and alleles of rs3740051 were estimated to compare differences between patients with PA and control subjects by gender. [file 12881_2019_892_MOESM2_ESM.docx]

***Additional file 2. The frequency of genotypes and alleles of rs3740051 in patients with PA and control subjects by gender***

| **Genotype/allele** | **Males** | | **p value*** | **Females** | | **p value*** |
| --- | --- | --- | --- | --- | --- | --- |
|  | **PA group, N (%) (n=55)** | **Control group, N (%) (n=320)** |  | **PA group, N (%) (n=87)** | **Control group, N (%) (n=506)** |  |
| Genotype  G/G  G/A  A/A  Allele  G  A | 1 (1.8)  10 (18.2)  44 (80.0)  12 (10.9)  98 (89.1) | 4 (1.2)  48 (15.0)  268 (83.8)  56 (8.8)  584 (91.2) | 0.779  0.466 | 1 (1.1)  12 (13.8)  74 (85.1)  14 (8.1)  160 (91.9) | 4 (0.8)  55 (10.9)  447 (88.3)  64 (6.3)  948 (93.7) | 0.682  0.397 |

*Pearson’s χ2 test, PA – pituitary adenoma, p value – significance level.
