# Supplementary material for: SIRT1 (rs3740051) role in pituitary adenoma development
Source: BMC Med Genet. 2019 Nov 20;20:185. doi: 10.1186/s12881-019-0892-x (PMC6868839; doi:10.1186/s12881-019-0892-x)
Supplement: Supplementary file 3 — Additional file 3. The impact of rs3740051 on PA development by gender. Logistic regression analysis was performed to evaluate the impact of rs3740051 on PA development under genetic models in female and male groups. [file 12881_2019_892_MOESM3_ESM.docx]

***Additional file 3. The impact of rs3740051 on PA development by gender***

| **Females** | | | | |
| --- | --- | --- | --- | --- |
| **Model** | **Genotype/allele** | **OR (95 % PI)** | **p value** | **AIC** |
| Codominant | G/A vs. A/A  G/G vs. A/A | 1.342 (0.685;2.629)  1.208 (0.139;10.487) | 0.391  0.864 | 497.796 |
| Dominant | G/A+G/G vs. A/A | 1.331 (0.696;2.547) | 0.388 | 495.804 |
| Recessive | G/G vs. G/A+A/A | 1.165 (0.134;10.095) | 0.890 | 496.499 |
| Overdominant | G/A vs. G/G+A/A | 1.339 (0.684;2.621) | 0.394 | 495.824 |
| Additive | G | 1.266 (0.714;2.246) | 0.420 | 495.898 |
| **Males** | | | | |
| Codominant | G/A vs. A/A  G/G vs. A/A | 1.269 (0.598;2.692)  1.523 (0.166;13.942) | 0.535  0.710 | 316.185 |
| Dominant | G/A+G/G vs. A/A | 1.288 (0.624;2.659) | 0.493 | 314.208 |
| Recessive | G/G vs. G/A+A/A | 1.463 (0.160;13.339) | 0.736 | 314.557 |
| Overdominant | G/A vs. G/G+A/A | 1.259 (0.594;2.668) | 0.547 | 314.312 |
| Additive | G | 1.258 (0.665;2.382) | 0.481 | 314.186 |

OR – odds ratio, CI – confidence interval, AIC-akaike information criteria, p-significance level.
