# Supplementary material for: SIRT1 (rs3740051) role in pituitary adenoma development
Source: BMC Med Genet. 2019 Nov 20;20:185. doi: 10.1186/s12881-019-0892-x (PMC6868839; doi:10.1186/s12881-019-0892-x)
Supplement: Supplementary file 4 — Additional file 4. The frequency of genotypes and alleles of rs3740051 in patients with active PA and inactive PA. Frequency of genotypes and alleles of rs3740051 were estimated to compare differences between patients with active PA and inactive PA. [file 12881_2019_892_MOESM4_ESM.docx]

***Additional file 4. The frequency of genotypes and alleles of rs3740051 in patients with active PA and inactive PA***

| **Genotype/allele** | **Frequency (%)** | | |
| --- | --- | --- | --- |
|  | **Active PA group, N (%) (n=80)** | **Non-active PA group, N (%) (n=62)** | **p value*** |
| Genotype  G/G  G/A  A/A  Allele  G  A | 1 (1.2)  11 (13.8)  68 (85.0)  13 (8.1)  147 (91.9) | 1 (1.6)  11 (17.7)  50 (80.6)  13 (10.5)  111 (89.5) | 0.472  0.494 |

*Pearson’s χ2 test, PA – pituitary adenoma, p value – significance level.
