# Supplementary material for: SIRT1 (rs3740051) role in pituitary adenoma development
Source: BMC Med Genet. 2019 Nov 20;20:185. doi: 10.1186/s12881-019-0892-x (PMC6868839; doi:10.1186/s12881-019-0892-x)
Supplement: Supplementary file 5 — Additional file 5. The frequency of genotypes and alleles of rs3740051 in patients with prolactinomas and control subjects. Frequency of genotypes and alleles of rs3740051 were estimated to compare differences between patients with prolactinomas and control subjects. [file 12881_2019_892_MOESM5_ESM.docx]

***Additional file 5. The frequency of genotypes and alleles of rs3740051 in patients with prolactinomas and control subjects***

| **Genotype/allele** | **Frequency (%)** | | |  |
| --- | --- | --- | --- | --- |
|  | **Control group, N (%) (n=826)** | **Prolactinomas group, N (%) (n=50)** | **p value*** |  |
| Genotype  G/G  G/A  A/A  Allele  G  A | 8 (1.0)  103 (12.5)  715 (86.6)  119 (7.2)  1533 (92.8) | 0 (0)  9 (18.0)  41 (82.0)  9 (9.0)  91 (91.0) | 0.397  0.503 |  |

PA – pituitary adenoma, p value – significance level, HWE p value – Hardy-Weinberg significance level

*Pearson’s χ2 test
