# Supplementary material for: SIRT1 (rs3740051) role in pituitary adenoma development
Source: BMC Med Genet. 2019 Nov 20;20:185. doi: 10.1186/s12881-019-0892-x (PMC6868839; doi:10.1186/s12881-019-0892-x)
Supplement: Supplementary file 6 — Additional file 6. The impact of rs3740051 on development of prolactinomas. Logistic regression analysis was performed to evaluate the impact of rs3740051 on prolactinomas development under genetic models. [file 12881_2019_892_MOESM6_ESM.docx]

***Additional file 6. The impact of rs3740051 on development of prolactinomas***

| **Prolactinomas** | | | | |
| --- | --- | --- | --- | --- |
| **Model** | **Genotype/allele** | **OR (95 % CI)** | **p value** | **AIC** |
| Codominant | G/A vs. A/A  G/G vs. A/A | 1.539 (0.726;3.260)  - | 0.261  0.999 | 385.193 |
| Dominant | G/A+G/G vs. A/A | 1.414 (0.669;2.989) | 0.364 | 384.654 |
| Recessive | G/G vs. G/A+A/A | - | 0.999 | 384.361 |
| Overdominant | G/A vs. G/G+A/A | 1.558 (0.736;3.301) | 0.247 | 384.390 |
| Additive | G | 1.242 (0.628;2.457) | 0.534 | 385.059 |

OR – odds ratio, CI – confidence interval, AIC-akaike information criteria, p-significance level.
